# Graded Maximal Exercise Testing to Assess Mouse Cardio-Metabolic Phenotypes

**DOI:** 10.1371/journal.pone.0148010

**Published:** 2016-02-09

**Authors:** Jennifer M. Petrosino, Valerie J. Heiss, Santosh K. Maurya, Anuradha Kalyanasundaram, Muthu Periasamy, Richard A. LaFountain, Jacob M. Wilson, Orlando P. Simonetti, Ouliana Ziouzenkova

**Affiliations:** 1 Department of Human Sciences, The Ohio State University, College of Education & Human Ecology, Columbus, Ohio, United States of America; 2 Cardiovascular Pathobiology Program, Sanford Burnham Medical Research Institute at Lake Nona, Orland, Florida, United States of America; 3 Department of Physiology and Cell Biology, The Ohio State University College of Medicine, Columbus, Ohio, United States of America; 4 Department of Human Performance, The University of Tampa, Tampa, Florida, United States of America; 5 Department of Radiology, The Ohio State University, College of Medicine, Columbus, Ohio, United States of America; 6 Department of Cardiovascular Medicine, The Ohio State University, College of Medicine, Columbus, Ohio, United States of America; 7 Biomedical Sciences Program, The Ohio State University, College of Medicine, Columbus, Ohio, United States of America; IGBMC/ICS, FRANCE

## Abstract

Functional assessments of cardiovascular fitness (CVF) are needed to establish animal models of dysfunction, test the effects of novel therapeutics, and establish the cardio-metabolic phenotype of mice. In humans, the graded maximal exercise test (GXT) is a standardized diagnostic for assessing CVF and mortality risk. These tests, which consist of concurrent staged increases in running speed and inclination, provide diagnostic cardio-metabolic parameters, such as, VO_2max_, anaerobic threshold, and metabolic crossover. Unlike the human-GXT, published mouse treadmill tests have set, not staged, increases in inclination as speed progress until exhaustion (PXT). Additionally, they often lack multiple cardio-metabolic parameters. Here, we developed a mouse-GXT with the intent of improving mouse-exercise testing sensitivity and developing translatable parameters to assess CVF in healthy and dysfunctional mice. The mouse-GXT, like the human-GXT, incorporated staged increases in inclination, speed, and intensity; and, was designed by considering imitations of the PXT and differences between human and mouse physiology. The mouse-GXT and PXTs were both tested in healthy mice (C57BL/6J, FVBN/J) to determine their ability to identify cardio-metabolic parameters (anaerobic threshold, VO_2max_, metabolic crossover) observed in human-GXTs. Next, theses assays were tested on established diet-induced (obese-C57BL/6J) and genetic (cardiac isoform *Casq2*^-/-^) models of cardiovascular dysfunction. Results showed that both tests reported VO_2max_ and provided reproducible data about performance. Only the mouse-GXT reproducibly identified anaerobic threshold, metabolic crossover, and detected impaired CVF in dysfunctional models. Our findings demonstrated that the mouse-GXT is a sensitive, non-invasive, and cost-effective method for assessing CVF in mice. This new test can be used as a functional assessment to determine the cardio-metabolic phenotype of various animal models or the effects of novel therapeutics.

## Introduction

Obesity rates are rising exponentially and increase patients’ risks for developing cardiovascular diseases [1]. Heart disease remains the leading cause of death in the United States and worldwide. Subsequently, this has stimulated a large interest in understanding the metabolic and physiological mechanisms regulating cardiovascular function and energy balance. For over 50 years, human research has used graded maximal exercise testing (GXT_h_) as the prototypical method to study cardiovascular and metabolic responses of the body to stress [2–4]. These standardized GXT_h_ tests, such as the gold standard Bruce protocol [5, 6], are key non-invasive and cost-effective methods for the assessing patient mortality risks [7, 8] and diagnosing coronary artery disease (CAD) [9].

New therapies for metabolic and cardiovascular disease have fast progressed with the development of genetic mouse models of cardiovascular dysfunction (reviewed in [10–14]). Genetic models, like the low-density lipoprotein receptor (*Ldlr*^***-/-***^) [11], apolipoprotein E (*ApoE*^-/-^) [15], and endothelial nitric oxide synthase (*eNos3*^-/-^) [16] knockout mice are well studied models of atherosclerosis. Similar to obese humans, diet-induced obese wild-type mice (WT-obese; C57BL/6J) can develop fatty streaks [17], left ventricular hypertrophy, and cardiac fibrosis [18–20]. Additionally, the WT-obese mouse model phenocopies the insulin resistant state, and subsequent impaired delivery of nutrients to skeletal muscles [21], that is seen in obese individuals during exercise testing [22]. Some knockout models are generated from mutations observed in patients with impaired cardiovascular function. These mouse models often phenocopy human mutations, develop cardiac dysfunction, and can be used to generate highly translatable findings. For example, the calsequestrin 2 (cardiac specific isoform) deficit mouse (*Casq2*^-/-^) phenocopies humans with CASQ2 mutations. Both *Casq2*^-/-^ mice and patients with CASQ2 mutations develop arrhythmias [23], catecholaminergic ventricular tachycardia, and can be diagnosed with exercise testing [24, 25]. Given the ability of mouse models to phenocopy various aspects of cardiovascular disease, the use of mouse models has become critical to the study of cardiac biology, physiology, and novel therapeutics prior to translating findings to man [10, 12–14, 26].

As a result of the expanding number of mouse models used to study metabolic and cardiovascular disease, animal exercise testing has become widely published [27–54] as a way to functionally characterize the cardiovascular fitness (CVF) of mice [55–58]. Maximal exercise testing is designed to induce specific stress to working muscles and the heart. During testing, cardiac output primarily drives the associated increase in oxygen consumption (VO_2_) until maximal oxygen consumption (VO_2max_) and exhaustion is achieved. This resulting state of VO_2max_ is characterized by sympathetic dominance, parasympathetic inhibition [59], and vasoconstriction to all systems but the heart, brain, and working muscles. While there are many conserved physiological responses between the cardiorespiratory systems of mice and men exposed to stress [60], there are also many differences that must be understood to enhance the interpretation and design of mouse exercise testing. The mouse heart is small (~0.2 g), beats between 400–600 beats per minute (bpm), and has a cardiac output (heart rate x strove volume) that is 2x-9x greater than humans. Differentially, the human heart is large (~250–300 g) and beats between 60–90 bpm at rest. Interestingly, when mouse and human stroke volume is normalized to bodyweight, there is not much discrepancy between the values recorded (reviewed in [13, 60, 61]). Many of the differences between human and mouse cardiac physiology are due to differences in heart size and rate, body mass, and oxygen requirements (reviewed in [60]). As a result, the information from exercise testing in mice and men will never be identical. Nonetheless, a lot of information can be generated from mouse treadmill tests with calorimetry data reporting, so long as the following established GXT_h_ considerations are adapted to mouse testing protocols:

1. modest stage to stage increases in energy requirements
2. a testing duration greater than 6 minutes
3. a test lasting no longer than 12 minutes [2, 62] and
4. appropriate acclimation of animals to treadmills.

Without these considerations, tests may lack the appropriate intensity needed to accurately assess the CVF of mice.

Currently, exercise testing in mice and men diverge in the areas of test design, time of test, and parameters that can be reported. GXT_h_ tests have staged concurrent increases in speed and inclination over the course of 8–12 minutes [9]. However, unlike with human testing, there are no standardized protocols or end point criteria for positive tests in mice. Most rodent assays are designed with increasing speed over a fixed inclination (defined as PXT_m_) for time periods greater than 12 minutes [23, 58, 63–69]. The most common variables reported in animal assays include VO_2max_, run time, and maximum run speed; which may not be sufficient for detection of impaired CVF [23]. Human testing differs in that it can derive additional diagnostic cardio-metabolic parameters such as anaerobic threshold (AT) [70], crossover (the shift from lipid to carbohydrate oxidation [71]), and pre- to post-test changes in lactate concentrations (Lactate_delta_) [72]. These variables provide valuable information regarding the ability of the cardiac and pulmonary systems to deliver oxygen (O_2_) during maximal and submaximal exercise intensities [73]. However, these parameters are rarely, if ever, reported in mouse testing because they cannot be accurately derived. Failure to derive these variables questions the ability of these assays to accurately assess mouse CVF. This, along with other limitations in currently utilized protocols (reviewed in [55, 74]), point out the need for a more efficacious and reliable standardized approach to test mouse CVF.

Here, we developed a new exercise testing method, the graded mouse maximal exercise test (GXT_m_) and describe how to derive novel diagnostic cardio-metabolic parameters in mice that can be generated from data acquired during maximal exercise testing. Additionally, we compare a new GXT_m_ to a PXT_m_, and the human GXT_h_. Our results showed that in mice, only the GXT_m_ was capable of generating cardio-metabolic parameters previously reported in human testing and consistently detecting impaired CVF in established mouse models of cardiovascular dysfunction.

## Methods

### Human Studies

#### Study Approval

For human testing, all subjects gave written informed consent prior to participation and all tests were done in accordance with procedures approved by The Ohio State University Biomedical Institution Review Board for this study. All animal experiments in this study were performed in accordance with procedures approved by Ohio State University Institutional Animal Care and Use Committee (IACUC) committee for this study and in accordance with the National Institutes of Health guidelines.

#### Human Bruce protocol testing (GXT_h_)

Healthy, recreationally trained men between 18 to 45 years of age were recruited from Columbus, Ohio to complete a Bruce protocol graded maximal treadmill exercise test following acclimation to test (*n* = 6). Testing was performed using ParvoMedics TrueOne 2400 systems and ParvoMedics software. ParvoMedics 2400 metabolic cart was turned on and allowed to warm up for at least thirty minutes prior to calibration and testing procedures. Pneumotachometer and gas analysis systems were calibrated according to manufacturer instructions before use during exercise testing. Expired gas was continuously sampled during exercise, through a 61cm Nafion tube (Permapure, Toms River, NJ, USA), via paramagnetic oxygen analyzer (0–25% range with 0.1% accuracy) and an infrared carbon dioxide analyzer (0–10% range with 0.1% accuracy). Metabolic data was sampled using 15 seconds averaging. Testing stages consisted of simultaneous increases in speed and inclination as previously described [6] (Table 1). All human subjects were required to achieve at least three of the following criteria indicating ${\dot{\text{V}}\text{O}}_{2}$max was reached: plateauing of oxygen consumption (${\dot{\text{V}}\text{O}}_{2}$), respiratory exchange ratio (RER) ≥ 1.1, heart rate ≥ 95% age-predicted maximal heart rate (APMHR), rating of perceived exertion (RPE) ≥ 17, respiratory rate (RR) > 40 breaths per minute, or subject inability to continue.

**Table 1:** VO_2max_ testing protocols in mice and men.

**PXT**_**m**_	**Speed**	**Incline**	**Duration**
Stage	(meter/min)	(% grade)	(min)
1	6	0	5
2	7	0	0.5
3	8	0	0.5
4	9	0	0.5
5	10	0	0.5
6	11	0	1
7	12	0	2
8	13	0	2
9	14	0	2
10	15	0	2
11	16	0	1
**GXT**_**h**_	**Speed**	**Incline**	**Duration**
Stage	(km/hr)	(% grade)	(min)
1	2.7	10	3
2	4	12	3
3	5.4	14	3
4	6.7	16	3
5	8	18	3
6	8.8	20	3
7	9.6	22	3
**GXT**_**m**_	**Speed**	**Incline**	**Duration**
Stage	(meter/min)	(% grade)	(min)
1	9	5	2
2	12	10	2
3	15	15	2
4	18	15	1
5	21	15	1
6	23	15	1
7	24.0+	15	1

Stages, speed, and incline of maximal exercise tests completed are described.

### Animal Studies

#### Animal subjects studied

Mice were housed with 12-hour light and dark cycles and maintained on a standard chow diet. C57BL/6J (WT, *n* = 7), FVB/NJ (Jackson Laboratories, Bar Harbor, Maine, *n* = 4), obese C57BL/6J on high fat diet (45kcal/fat, Research Diets Inc, New Brunswick, NJ, ~100 days of high fat diet feeding, *n* = 11) and Calsequestrin 2 (cardiac isoform) null (*Casq2*^*-/-*^, *n* = 4)(*Mus musculus*) [23, 75] male mice, 4–6 months old, were used. Metabolic and physiological parameters are described in S1 Table.

#### Animal acclimatization to treadmills

Animals were first acclimated (S2 Table) to the treadmill (Metabolic Modular Treadmill; Columbus Instruments, Columbus, OH, USA) and then rested for one week prior to performing the GXT_m_. Acclimation consisted of 3 training sessions with 60 hours recovery between sessions. During acclimation mice were placed in a motionless treadmill for 3 minutes, after which the shock grid was activated (3 Hz and 1.5 mA). Next, the treadmill was engaged to a walking speed of 6 m/min for 5 minutes and progressively increased up to 12 m/min for a total duration of 12 minutes of exercise.

#### Software calibration and calculations from Metabolic Modulator Treadmill

Before each testing session, Oxymax software (Columbus Instruments, Columbus, OH, USA) and open circuit indirect calorimetry treadmills (Metabolic Modular Treadmill, Columbus Instruments, Columbus, OH) [76] were calibrated and checked for hardware malfunctions according to manufacturer instructions. Prior to calibration, sample pump was turned on with flow indicator showing flow set at 4–5 LPM. Pressure reading was set at ~800mmHg and gas tank output pressure was set at 10psi. Gas calibration was performed and adjusted when necessary using the GAIN and FINE knobs to set reading at 0.50% CO_2_ and 20.5% O_2_. Drierite (Calcium Sulfate with Indicator, Sigma-Aldrich; St. Louis, MO, USA) was changed constantly to maintain accurate gas readings and to assure that moisture accumulating during testing could properly be absorbed. During testing, analysis was set to collect gas exchange measures every 15 seconds (settings: cage settle was set to every 15 sec; cage measure was set to every 15 sec; reference settle was set to every 30 sec; reference measure was set to every 30sec, volume rate unit was set to ml/kg/min, and accumulated gas unit was set as liter). During experiments, system sample pump maintained a constant sample flow reading of 0.5 L/min and sample drier a purge gas flow reading of 1.5 L/min. Maximum run speed (meter/min), shock grid contact (seconds) and time until exhaustion (min) were manually recorded with stopwatch. Oxymax computer software collected gas concentrations and flow to calculate oxygen consumption (VO_2_), carbon dioxide expiration (VCO_2_), and RER (VCO_2_/VO_2_) from the treadmill every 15 sec. Oxymax gas exchange calculations and generation of RER derived fuel substrate oxidation are additionally listed in the supplementary materials (S2 Text, S3 Table).

#### Mouse Graded Maximal Exercise Test (GXT_m_)

Following one week of rest from acclimation training, mice were placed on the treadmill at 0° incline and the shock grid was activated. The treadmill speeds were then increased until exhaustion as follows: (speed, duration, grade)—(0 m/min, 3 min, 0°), (6 m/min, 2 min, 0°), (9 m/min, 2 minutes, 5°), (12m/min, 2 min, 10°), (15m/min, 2 min, 15°), (18, 21, 23, 24 m/min, 1 min, 15°), and (+1 m/min, each 1 min thereafter, 15°). Exhaustion (endpoint for treadmill cessation) was defined as the point at which mice maintained continuous contact with the shock grid for 5 seconds. Continuous contact is defined as any portion of the animal’s body coming in contact with the shock grid for a total of 5 seconds. During the test, occasional (~1–5 times per single animal test) 1–2 second tail contacts were observed when animals misstepped or were slow to response in the increase in intensity. VO_2max_ was determined by the peak oxygen consumption reached during this test when RER was >1.0. Maximum running speed was defined as the treadmill speed at which VO_2max_ was achieved (Table 1). All animals (within-subjects design, GXT_m_ and PXT_m_) underwent pre- and post-test lactate assays (Lactate assay) one hour prior to and immediately following exercise testing)**.**

#### Mouse Progressive Maximal Exercise Test (PXT_m_)

Following one week of rest after GXT_m_ the PXT_m_ was conducted as described in [56]. Specifically, mice were placed on the treadmill (0° incline entire experiment) and the shock grid was activated. The treadmill speeds were then increased until exhaustion as follows: (speed, duration)—(0 m/min, 5 min), (6 m/min, 5 min), (7, 8, 9, and 10 m/min, 30s each), (11m/min,1 min), (12, 13, 14, and 15 m/min, 2 min each), and (+1 m/min, each 1 min thereafter). Exhaustion (endpoint for treadmill cessation) was defined as the point at which mice maintained continuous contact with the shock grid for 5 seconds (further described in the GXT_m_ section). VO_2 max_ was determined by the peak oxygen consumption reached during this test when RER was ≥ 1.0. Maximum running speed was defined as the treadmill speed at which VO_2max_ was achieved. All animals (within-subjects design, GXT_m_ and PXT_m_) underwent pre- and post-test lactate assays one hour prior to and immediately following exercise testing)**.**

#### Lactate assay

A protocol [77] was adapted to measure venous blood lactate concentrations from the tail vein. During acclimation exercise sessions, mice were also acclimated to tail vein blood collection (3 pre acclimation session collections, and 3 post acclimation session collections). For the PXT_m_ and GXT_m_; 1 hour prior to testing, ~0.7μL of blood (via tail vein prick) was collected and placed for analysis on a handheld lactate meter (Lactate Plus; Nova Biomedical, Waltham, MA, USA). Within one minute of test completion, ~0.7μL of blood was again collected and analyzed. For all testing, the same device was utilized to reduce variability.

#### Statistical Analysis

Data processing: Prior to analysis, the dependent variables with the four genotypes (WT, WT obese, *Casq2*^*-/-*^, FVB/NJ) and two test types (GXT_m_, PXT_m_) were examined through IBM SPSS version 22 (9.5.0.0) for accuracy of data entry, fit between their distributions, and the assumptions of multivariate analysis. Upon inspection of standardized scores, there were no univariate outliers. Mahalanobis distance values were requested and no multivariate outliers were identified as exceeding the Mahalanobis distance value at p < .01 (χ^2^ = 32.00, *df* = 16, *p* = .01). Therefore, no additional cases were removed from the dataset. A review of plots of the residuals for each of the five dependent variables by group indicated that the assumption of independence was satisfied. Pairwise linearity was checked to determine the relationship between dependent variables using within-group scatterplots and also found to be satisfactory. All skewness and kurtosis statistics were between the range of -2 and 2, providing evidence that normality was a reasonable assumption. Further evidence of normality can be seen through the visible inspection of Q-Q plots and histograms of each dependent variable. There were no issues with normality observed.

Analyses and statistical tests: were performed with IBM SPSS Statistics 22 (9.5.0.0). All values represent mean SD unless noted otherwise. Two-Three group MANOVAS were performed. The Bonferroni correction when applied to an alpha of .05 yielded an alpha level of .007 for the univariate ANOVAs and Tukey HSD Multiple Comparisons presented. When appropriate, Student's two-tailed *t*-test were applied with *P*-values <0.05 being considered significant. The results of the ANCOVA tests, with weight as a covariant, for the calorimetry data (VO_2_ data) and information regarding statistical test selection for calorimetry data analysis is further described in S3 Text.

## Results

### Development of a GXT_m_ exercise assay for mice

Our goal was to develop a test for mice that provided cardio-metabolic parameters previously reported in the human GXT_h_ and to compare those parameters describing mouse performance during both the GXT_m_ and PXT_m_. We used lean WT (C57BL/6J) male mice as a control group, C57BL/6J male mice with diet induced obesity (WT-Obese) as a non-transgenic model of cardiac deficiency [18–20, 78], and calsequestrin 2 (cardiac isoform) deficient (*Casq2*^-/-^) male mice as a genetic model with reported cardiac deficiency [23, 75] (S1 Table). During the study, no animals experienced adverse effects from exercise testing that required them to be removed early from testing.

Within-subjects design for PXT_m_ and GXT_m_ was used to reduce errors associated with individual differences. Examples of representative single tests and the averaged measurements of all tests are shown in S1 and S2 Figs. Initially, we performed the GXT_m_ and the PXT_m_ using control WT male mice and the GXT_h_ using healthy male human subjects. For all protocols, both mouse and human subjects underwent acclimation prior to testing. In mice, following the GXT_m_, an additional week of rest was given before animals performed the PXT_m_. This was done to minimize the effect of training induced adaptations. Of note, we performed the testing in opposite order and found no differences in performance (data not shown). Additionally, all mice underwent pre- and post-test lactate (LA) assays one hour prior to and following test termination (~within one minute of treadmill stopping and animal being removed) as biochemical confirmation that exhaustion was achieved.

**Fig 1:**
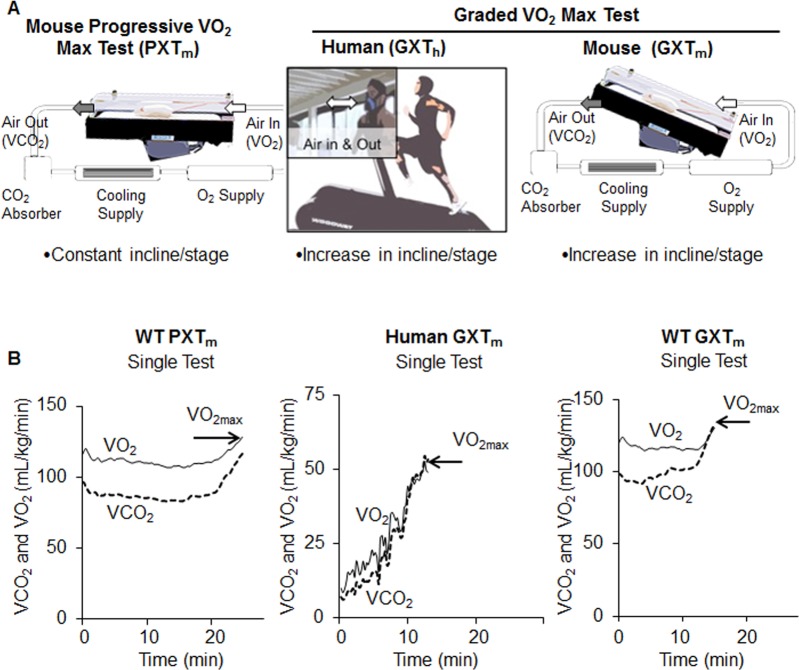
Description of exercise testing in mice and men. (A) Schematic description of exercise testing in mice and men. The PXT_m_ maintains fixed inclination (0°) while speed increases until the test is terminated (Table 1). Training in mice was done on a chamber-enclosed treadmill that allowed it to function as an open circuit indirect calorimeter; and thus, allowed for derivation of VO_2_ and VCO_2_ values. With the GXT_m_ (middle) and GXT_h_ (right), speed and incline simultaneously increased as stages progressed (Table 1). (B) Human and mouse tests used indirect calorimetry to measure VO_2_ (solid line) and record VO_2max_ as well as measure CO_2_ (dashed line). Mouse and man tests were randomly selected from WT males (*n* = 7) and healthy men *(n* = 6) and used for derivation of all parameters (all data are shown in S1 and S2 Figs). During maximal exercise testing both species have similar responses (RER, lactic acid formation, fuel utilization, O_2_ use, heart rate, speed, exhaustion). In the GXT_m_, and GXT_h_. at VO_2max_, VCO_2_ intersected or surpassed VO_2_, and was a parameter of a positive test (as RER >1.0, middle and right panel). In the PXT_m_, VO_2max_ did not fulfill this criterion (left panel).

We developed the GXT_m_ protocol taking into consideration that most PXT_m_ tests [23, 58, 63–69, 79–83] increase the speed at a set incline over time (Table 1, Fig 1A) until maximal exertion and a respiratory exchange ratio (RER, the quotient of (VCO_2_/ VO_2_) ≥ 1.0 is achieved (S4 Table)**.** VO_2max_ testing is limited by the ability of the cardiorespiratory system to supply oxygen to working muscles. Experiments altering oxygen delivery (hypoxia), overperfusing muscles during exercise, and showing the contributions of cardiac output relative to arterial-venous oxygen (reviewed in [84]) have been key in determining this concept. Accordingly, both the human and mouse GXT were designed to promote an end stage in which VO_2_ fails to rise as oxygen demands increase. This end state is marked by predominately cardiac fatigue and an enhanced dependence on anaerobic glycolysis [84] prior to the onset of skeletal muscle exhaustion.

In our human GXT_h_ and established GXT_h_ protocols (Table 1), there are simultaneous staged increases in speed and incline until the following conditions are met [9]: 1) maximal exertion, 2) achievement of RER ≥ 1.1, 3) a plateau or decrease following peak oxygen consumption, 4) a significant increase in pre- to post-test venous blood LA concentrations (~8-10mmol/l), and 5) failure of heart rate to increase with increasing exercise intensity (S4 Table). We developed a similar, but not identical, GXT test in mice (GXT_m_) by have stages of simultaneous increases in speed and incline to achieve: 1) maximal exertion, 2) achievement of RER ≥ 1.0, 3) a plateau or decrease following peak oxygen consumption, and 4) a significant increase in post-test venous LA concentrations (~8mmol/l). Treadmill inclination increases, which were restricted to 5° increments, were capped at 15° due to observations in initial method development which showed that mice struggled to maintain natural gait with incline set >15°. The end points of all exercise tests are described in S5 Table. In mice, maximal exertion on the test was measured as time until exhaustion (minutes), and determined by ≥ 5 seconds of continuous contact with the shock grid. Continuous contact was defined as any portion of the animal’s body coming into contact with the shock grid. It should be noted that rarely were animals seen sitting down on the shock grid. Instead, most continuous contacts consisted of the animal’s tail or hind limb partially contacting the shock grid. Exhaustion was further validated using biochemical measures of circulating LA concentrations.

### Reported measures in healthy mice and man during exercise testing

Oxygen consumption (VO_2_) and carbon dioxide expiration (VCO_2_) were two principal measures obtained from the metabolic treadmill (S1 and S2 Texts) during testing. In healthy WT mice, both the PXT_m_ and GXT_m_ showed increases in VO_2_ and VCO_2_ during testing (Fig 1B); however, in the majority of single PXT_m_ tests_,_ VCO_2_ and VO_2_ did not intersect at VO_2max_. This suggested that true maximum was not achieved. In the GXT_m,_ all single tests showed a clear intersection of VCO_2_ and VO_2_.

Time until exhaustion lasted for 20 to 29 minutes with PXT_m_ excluding warm-up (Fig 1B). The most commonly used GXT_h_, the Bruce protocol, elicits time until exhaustion between 8–12 minutes in the general population [4, 5, 9]. Similarly to the reported data, our GXT_h_ and GXT_m_ tests achieved exhaustion between 8–12.5 minutes in WT mice and healthy humans (excluding warm-up) (Fig 1B). Furthermore, the GXT_m_ data consistently produced VO_2max_ values that were accompanied by exhaustive efforts and increased blood LA concentrations. Significant elevation of blood lactate post-test is a marker for the transition from aerobic to anaerobic metabolism and only consistently occurred in GXT_m_ (data for all mouse groups are shown and discussed later).

**Fig 2:**
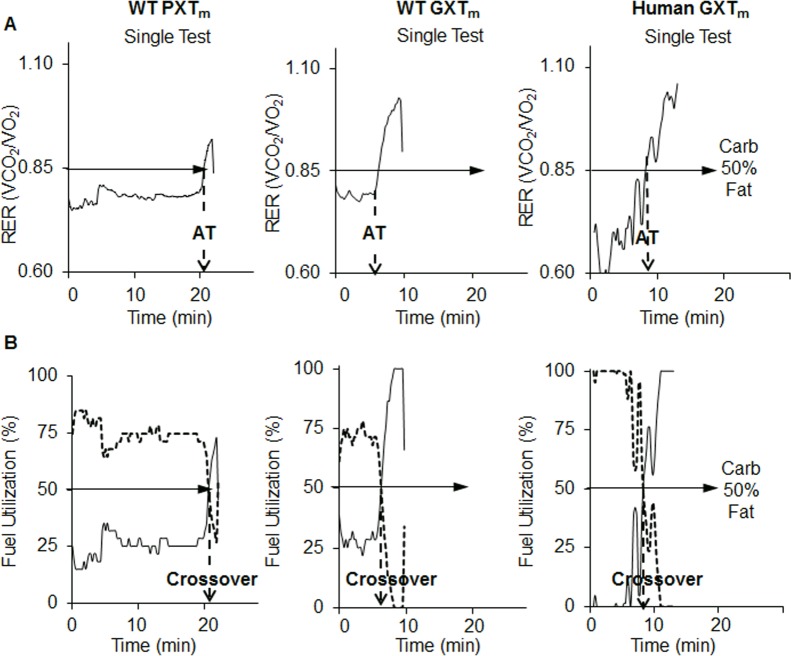
Kinetics and parameters from PXT_m_, GXT_m_, and GXT_h_ using single test analysis. The same single mouse and man tests were randomly selected from WT males (*n* = 7) and healthy men *(n* = 6) and used for derivation of all parameters. (A) RER (VCO_2_/ VO_2_) represents fuel substrate utilization during PXT_m_, GXT_m_, and GXT_h_. An RER of .85 (solid horizontal arrow) indicates 50% carbohydrate and 50% fat oxidation. In a single test, the point in which there is an abrupt increase in RER is known as anaerobic threshold (AT, dashed arrow). (B) Carbohydrate (dashed line) and fat (solid line) oxidation was determined from RER values (S6 Table) in the PXT_m_, GXT_m_, and GXT_h_. Dotted vertical arrow indicates the crossover time point in the test where there is a shift from predominant lipid oxidation to predominate carbohydrate oxidation.

RER was used to determine anaerobic threshold (AT) and fuel substrate (carbohydrate and lipid) oxidation (S3 Table) during testing. Anaerobic threshold (AT) is the point at which there is a shift from aerobic to anaerobic metabolism and signifies the onset of metabolic acidosis during continuous exercise [85]. The standard method for determining AT in humans is through multiple blood draws while running. This determination method was not feasible to execute in mice constrained in an enclosed metabolic treadmill. Thus, we determined AT using the method of identifying an abrupt increase in RER kinetics [85] and were able to consistently determine AT from RER kinetics in both human and mouse GXT single tests (Fig 2A).

AT was more difficult to consistently determine from single PXT_m_ tests, and in those where it could be determined, it occurred approximately 20 minutes or longer into the test compared to the GXT_m_. Using RER values, we were also able to calculate a previously established GXT_h_ parameter known as the crossover point (the transition from fat to carbohydrate oxidation [71]). Each single GXT_m_ test and averaged test allowed for crossover determination (Fig 2B); however, a specific crossover point could not be determined from most single PXT_m_s (Fig 2B). Crossover could be determined from the averaged PXT_m_ (Fig 2B). In the averaged GXT_m_, compared to the averaged PXT_m_, crossover occurred sooner and at a similar time point to the GXT_h_ (S1 Fig). Of note, cardio-metabolic parameters (VO_2_, VCO_2_, RER, AT, crossover) were derived from single tests, and then averaged, when completing analysis. Our findings indicated that longer tests with progressive intensity increases, like the PXT_m,_ were not capable of producing VO_2max_ with associated biochemical increases in lactate and parameters specific to an increased reliance on the glycolytic system (crossover, AT). These measures were; however, reported in both healthy mice and men during the GXTs.

### Sensitivity of mouse testing methods to detect impaired cardiovascular fitness

**Fig 3:**
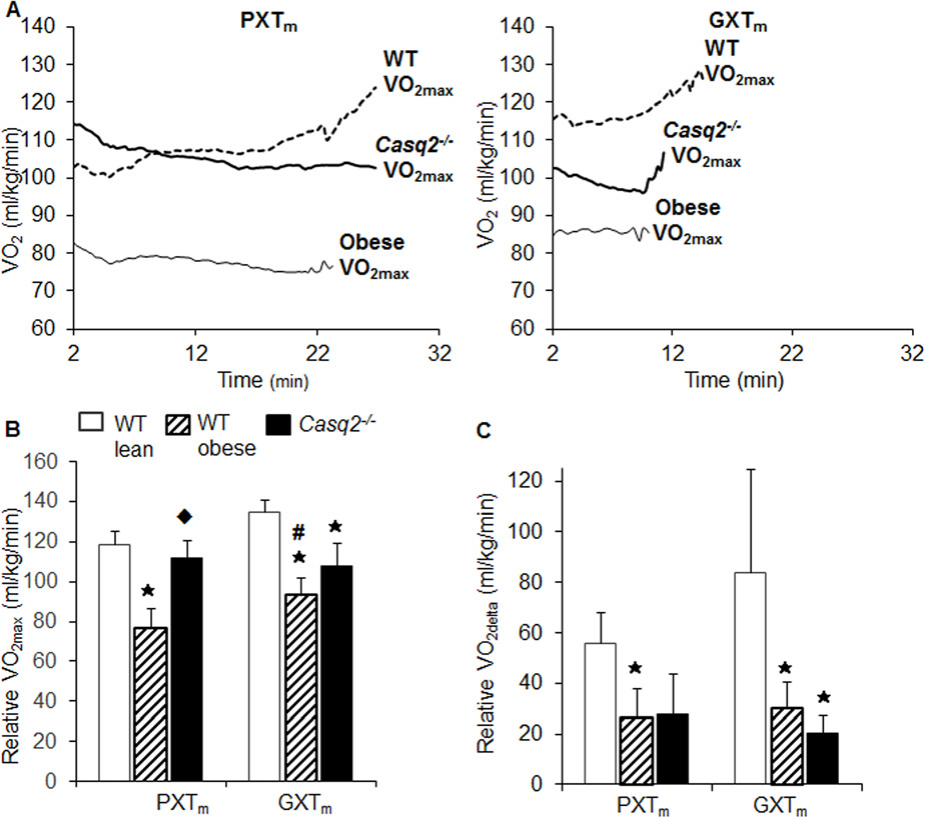
VO_2max_ achieved with the GXT_m,_ but not the PXT_m,_ identifies impaired cardiovascular fitness in mouse models of cardiovascular dysfunction. (A) Averaged VO_2_ kinetics obtained from WT (dashed line), *Casq2*^-/-^ (solid thick line), and obese (solid line) mice that performed the PXT_m_ and GXT_m_. VO_2_ is indicated for each group from the beginning of the test until the end (Minute 2, after the 2 minute warm, to point of exhaustion). Of note, during the PXT_m_, the largest increase in VO_2_ occurred with the first stage, and leveled off as the test continued in dysfunctional models. (B) Relative VO_2max_ values and (C) change from baseline to maximal oxygen consumption (VO_2delta_) are compared (mean±SD, MANOVA, Tukey HSD Multiple Comparisons, alpha = .007) in WT-healthy (white bar), WT-obese (hashed bar), and *Casq2*^-/-^ (black bar) mice. Asterisks indicate significance at the alpha = .007 level (MANOVA, multiple comparisons Tukey HSD for the PXT_m_ and GXT_m_ of WT v. obese and WT v. *Casq2*^-/-^), hash indicates significant difference at the alpha = .05 level between tests for the same genotypes (Student’s t-Test), and diamond indicates significance at the alpha = .007 level (MANOVA, multiple comparisons Tukey HSD for the PXT_m_ and GXT_m_ of obese v. *Casq2*^-/-^).

Next, we quantitatively compared PXT_m_ and GXT_m_ tests in dysfunctional mouse models to assess their sensitivity in detecting impaired levels of CVF. Averaged kinetics for VO_2_ revealed differences among WT-lean, WT-obese, and *Casq2*^*-/-*^ mice with the GXT_m_. With the PXT_m_, VO_2_ kinetics was similar between the *Casq*2^-/-^ and obese mice; with both strains failing to show a progressive increase in VO_2_ over the course of the test (Fig 3A). Only the WT mice showed increases in VO_2_ as the PXT_m_ progressed. With the PXT_m_, relative VO_2max_ (VO_2max_ normalized to body weight) was only significantly suppressed in the obese group. Additionally, VO_2max_ was unchanged between the WT and the *Casq2*^*-/-*^ (Fig 3B)**.** With the GXT_m_, relative VO_2max_ was significantly suppressed in both dysfunctional models (alpha = .007, MANOVA, Tukey HSD Multiple Comparisons; *p* < .001, WT v. obese; *p* = .001; WT v. *Casq2*^*-/-*^; *p* = .001 obese v. *Casq2*^*-/-*^; Fig 3B). Of note, an expected increase from basal VO_2_ to VO_2max_ (VO_2delta_) was achieved in all tests (PXT_m_, GXT_m_) with the exception of WT v. obese using the PXT_m_ (alpha = .007, MANOVA, Tukey HSD Multiple Comparisons; *p =* .006, WT v. obese; Fig 3C). However, this could have been observed as a result of the PXT_m_ eliciting a smaller VO_2delta_ in WT compared to the GXT_m_ (*p* < .05, Student’s t-Test). We validated all data with an additional control strain (S5 and S7 Tables) to further confirm the sensitivity of each test.

**Fig 4:**
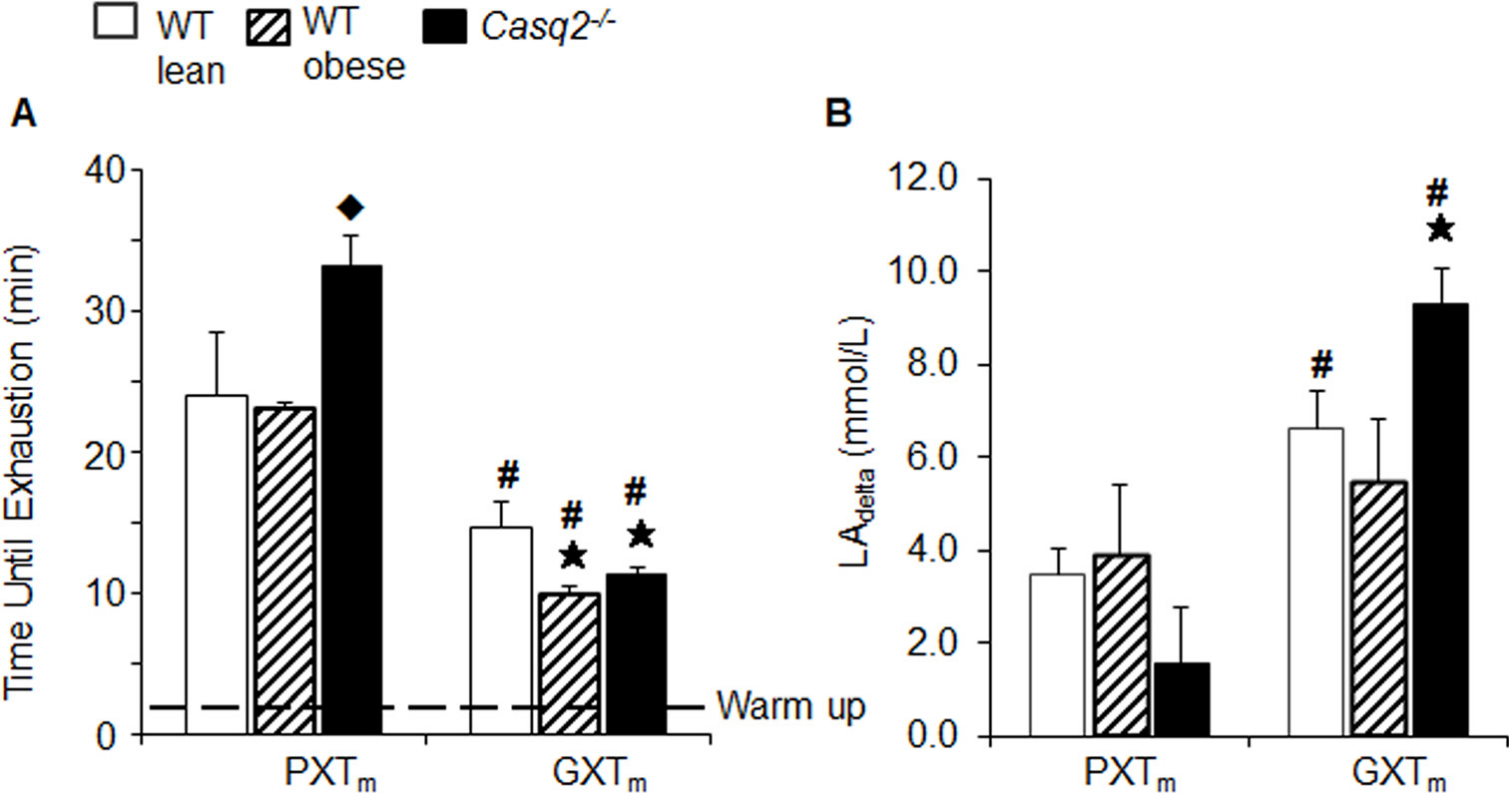
Time until exhaustion at true VO_2max_ is confirmed by increases in lactate concentrations and demonstrates impaired cardiovascular fitness with the GXT_m,_ but not the PXT_m_. (A) Time until exhaustion derived from VO_2_ kinetics in same groups of mice described in Fig 3. The dashed line shows the time used for warm up. (B) Change from pre to post test lactate concentration (LA_delta_) in same group of mice. Bar graphs represent mean ± SD for all but lactate, which is mean ± SEM. Student’s t-Test; *p* < .05, WT v. *Casq2*^-/-^.

**Fig 5:**
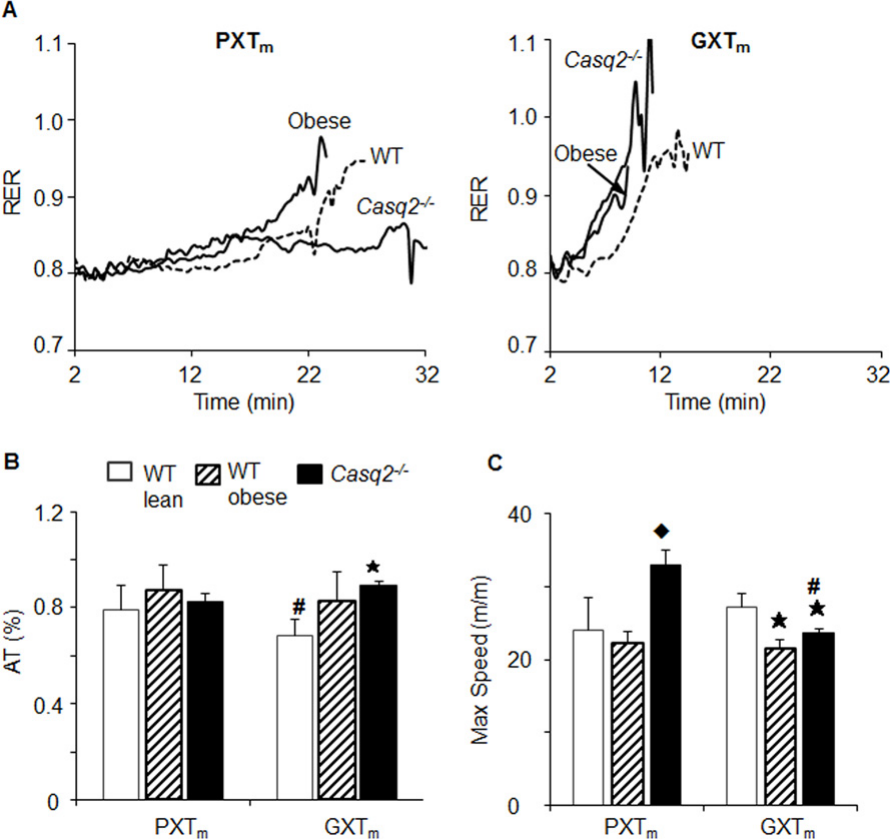
Anaerobic threshold and maximum speed assess dysfunction in mice with the GXT_m_, but not the PXT_m_. (A) Average RER kinetics from same from WT (dashed line), *Casq2*^-/-^ (solid thick line), and obese (solid line) mice performed the PXT_m_ and GXT_m_. (B) AT was reported as %AT, a time point where AT occurred/total test time in mouse groups (Fig 3). (C) Maximum speed achieved on test (m/m). For all measures, asterisks indicates significance at the alpha = .007 level (MANOVA, multiple comparisons Tukey HSD for the PXT_m_ and GXT_m_ of WT v. obese and WT v. *Casq2*^-/-^), hash indicates significant difference at the alpha = .05 level between tests for the same genotype (Student’s t-Test), and diamond indicates significance at the alpha = .007 level (mean ± SD, MANOVA, multiple comparisons Tukey HSD for the PXT_m_ and GXT_m_ of obese v. *Casq2*^-/-^).

Similar to VO_2max_ data, only the GXT_m_ provided a significant decrease in time until exhaustion in the *Casq2*^*-/-*^ and obese mice (alpha = .007; MANOVA, Tukey HSD Multiple Comparisons; *p* = .006, *Casq2*^*-/-*^; *p* = .001, obese; Fig 4A). Maximum run speed was also only significantly reduced with the GXT_m_, but not the PXT_m_, in both dysfunctional models (alpha = .007; MANOVA, Tukey HSD Multiple Comparisons; *p* = .006, *Casq2*^*-/-*^; *p* = .001, obese; Fig 5C). In the PXT_m_, significant reductions in time until exhaustion and run speed were not observed in the dysfunctional groups (Fig 4A and Fig 5C). This indicated that the PXT_m_ did not induce sufficient cardiovascular stress to allow for the detection of impaired CVF in established models of cardiac insufficiency. The results of the ANCOVA tests with weight being used as a covariant for the calorimetry data (VO_2_ and other variables) are also described and discussed in S3 Text.

The *Casq2*^*-/-*^ mice, a known model of cardiac insufficiency [23] ran longer than healthy WT controls during the PXT_m_ (Fig 4A, S4 Text). This was not seen with the GXT_m_ though; as the WT performed the longest and ran the fastest (Fig 5A). Considering that it has already been established that the *Casq2*^*-/-*^ model phenocopies humans with CASQ2 mutations [23], and that humans with CASQ2 mutations can be diagnosed with graded maximal exercise tests, we performed further studies investigating the performance of the *Casq2*^*-/-*^ mice [23, 86–88] (S4 Text, and S8 Table). We concluded that *Casq2*^*-/-*^ had superior performance on the PXT_m,_ but impaired performance on the GXT_m,_ because the PXT_m_ did not provide enough stress to elicit impaired CVF in these mice. That conclusion was in line with the original findings that showed running time until exhaustion does not change between WT and *Casq2*^*-/-*^ mice when the maximal exercise test has a set inclination. It should be noted, that both catecholamine challenge and exhaustive exercise with ECG monitoring have shown that *Casq2*^*-/-*^ mice display cardiovascular dysfunction in the form of catecholaminergic polymorphic ventricular tachycardia [23].

The onset of exhaustion is validated in human testing by elevated post-test blood LA (LA_delta_; LA_post_-LA_pre_) concentrations (~8-10mmol/) compared to baseline [4, 55, 74]. We observed significant increases in LA_delta_ using the GXT_m_ compared to the PXT_m_ in WT and *Casq2*^*-/-*^ groups. With the GXT_m_, *Casq2*^-/-^ mice had significantly greater LA_delta_ compared to WT; however, with the PXT_m,_ this parameter was decreased compared to controls (Fig 4B, S9 Table). In humans with myocardial ischemia, a hallmark response to a GXT_h_ is a significant increase in circulating blood LA concentrations compared to healthy subjects [89]. Thus, this response was replicated with the GXT_m_ in the genetic model of cardiac insufficiency (9.32 ±1.53mmol/L, *Casq2*^-/-^ v. 6.63 ±17mmol/L; WT; Student’s t-Test, alpha = .05; Fig 4B, S9 Table).

RER kinetics indicated that PXT_m_ could not clearly determine AT to assess CVF; however, RER kinetics from all single GXT_m_ was capable of determining AT in healthy and dysfunctional models. No significant difference was found between the mean relative AT in functional and dysfunctional mice using the PXT_m_ (alpha = .007, MANOVA, Tukey HSD Multiple Comparisons, *p* = .002; WT v. *Casq2*^-/-^ during GXT_m_; Fig 5B). With the GXT_m_, *Casq2*^-/-^ mice also had significantly higher relative ATs compared to WT controls. Thus, with GXT_m_, AT was lower in dysfunctional mice; a finding similar to those from human research looking at AT in patients with cardiac disease above functional class I (S5 Text, [90]). Together, these results demonstrated that the GXT_m_, like the GXT_h_ [4, 72, 74, 89, 91], was able to simultaneously elicit exhaustive efforts, true VO_2max_, and shift to anaerobic metabolism [4, 72, 74, 89, 91].

### Fuel utilization differs between healthy mice and models of cardiovascular dysfunction during exercise testing

**Fig 6:**
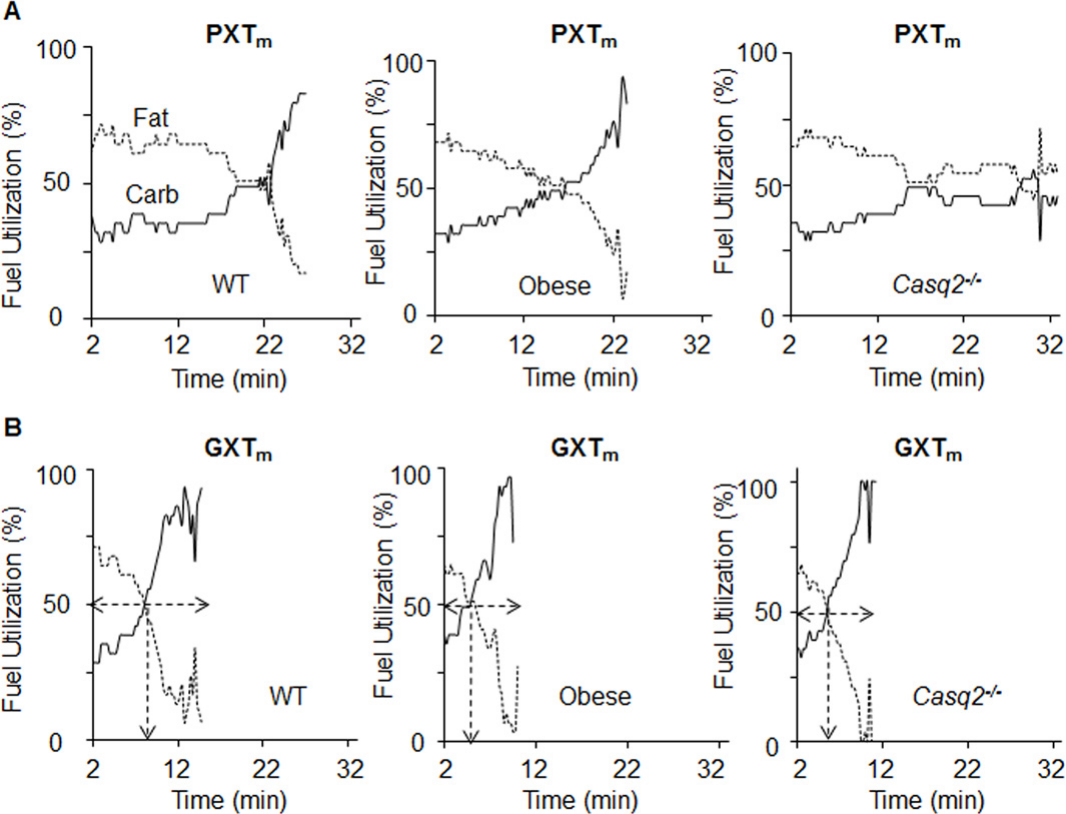
Carbohydrate and fat oxidation kinetics can be used to identify the crossover point in the GXT_m_, but not the PXT_m_. Averaged fuel utilization kinetics in WT (*n* = 7), obese (*n* = 11), and *Casq2*^-/-^ (*n* = 4) mice. Fat (dashed line) and carbohydrate (Carb, solid line)) oxidation were derived from RER as described in S3 Table during the PXT_m_ (A) and GXT_m_ tests (B). In GXT_m_ tests, the arrow indicates crossover, the point at which carbohydrate and fat oxidation intersect (dashed arrows).

We determined values of carbohydrate or fat oxidation during both the PXT_m_ and GXT_m_ by converting RER values recording during testing into their respective fat and carbohydrate oxidation values (Fig 6, S3 Table).

**Fig 7:**
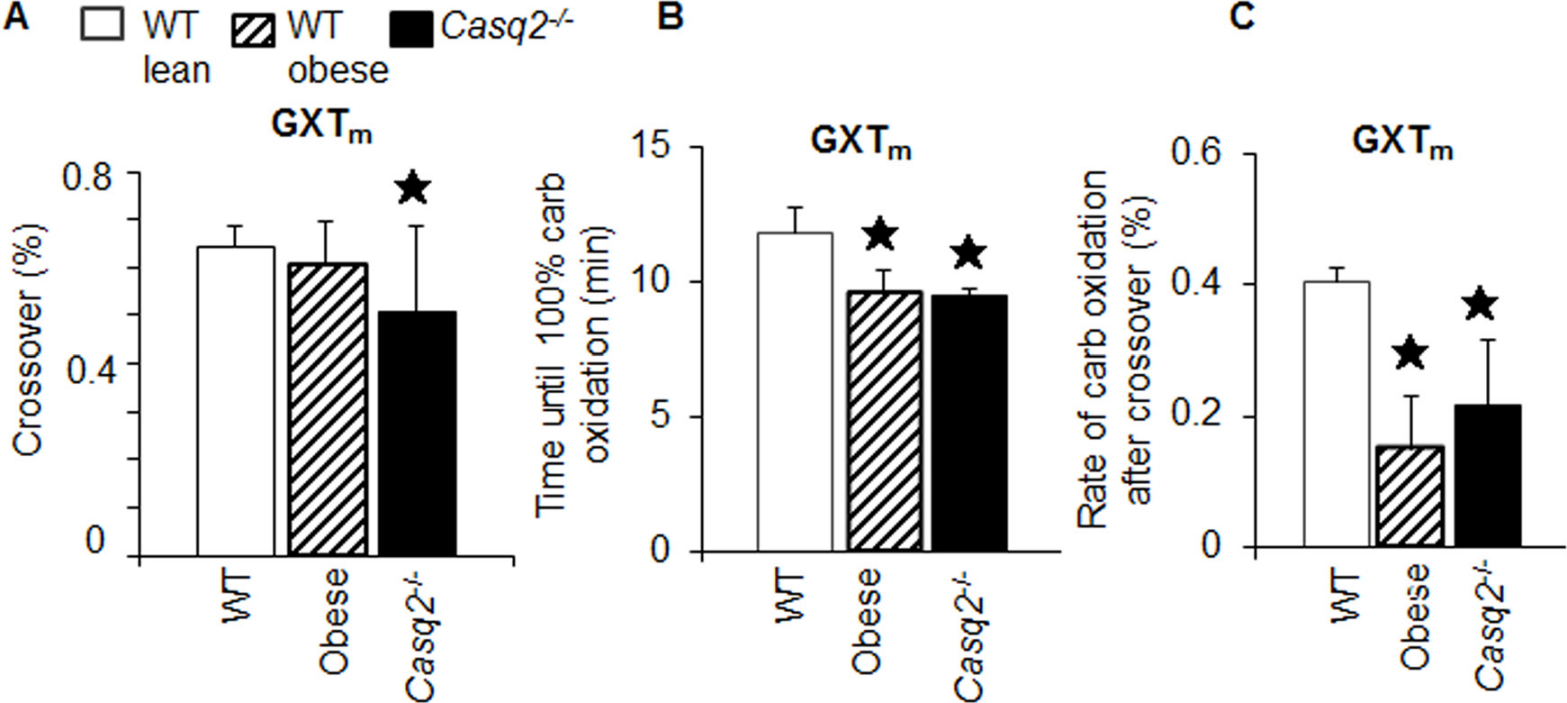
Carbohydrate and fat oxidation parameters from the GXT_m_, but not the PXT_m_, can be used to identify impaired cardiovascular fitness in dysfunctional models. (A) Fuel utilization kinetics (Fig 6B) from the GXT_m_ were used to quantify the percent of the test at which the crossover point was achieved relative to total time of test (% of test time at which crossover is achieved is the minute crossover occurred divided by total test time and multiplied by 100). Asterisk indicates significance at the alpha = .007 level (MANOVA, multiple comparisons Tukey HSD for the GXT_m_). (B) Time until 100% carbohydrate oxidation. Asterisks show significant difference between dysfunctional v. WT groups, (Student’s T-test, GXT_m_ for WT v. obese and WT v. *Casq2*^-/-^). (C) Rate of carbohydrate oxidation after crossover in all mouse groups. This rate is determined by dividing time after crossover by total time of test (100%) (Student’s T-test for the GXT_m_ for WT v. obese and WT v. *Casq2*^-/-^. Bar graphs represent mean ± SD).

With PXT_m_ there were multiple crossover points from fat to carbohydrate oxidation during tests, making it difficult to identify a single crossover point. Unlike the PXT_m_, the GXT_m_ allowed for identification of an accurate crossover point in all single tests. In humans, crossover occurs at between 60–80% of aerobic power [71]. We observed crossover in this range with all genotypes on the GXT_m_. Specifically, it occurred sooner in the *Casq2*^-/-^ compared to WT controls (Student’s T-test, *p* < .05; WT v. *Casq2*^-/-^; Fig 7A). Time to 100% carbohydrate oxidation during GXT_m_ testing was significantly shorter in dysfunctional animals compared to WT mice (Student’s T-test, *p* < .05; WT v. *Casq2*^-/-^, *p* < .05; WT v. obese; Fig 7B) and the rate of carbohydrate oxidation after crossover was decreased (Student’s T-test, *p* < .05; WT v. *Casq2*^-/-^, *p* < .05; WT v. obese; Fig 7C). Together these results indicated that substrate utilization parameters from the GXT_m_ could be used to identify the crossover point and determine fuel use during aerobic and anaerobic stress conditions.

## Discussion

### GXT design considerations

For over 50 years, exercise testing has served as an established and validated method for diagnostic and prognostic assessment of CVF in the clinical setting [9]. Physicians and exercise physiologists value the use of the GXT_h_ for induction of physiological stress [92] to the cardiopulmonary system in a controlled environment with simultaneous monitoring of myocardial oxygen demands [93], biochemical [94], and metabolic [71] responses. Furthermore, it is a validated method of evaluating the status of patients with cardiovascular and pulmonary disease [2]. Established GXT_h_ tests involve gradual increases in work output over multi-stage increases in speed and inclination [95] (Table 1). When generating the GXT_m_, we applied the same principle design and used staged increases in speed and inclination, as well as similar end point criteria for a positive test (S4 Table). Since both human and mouse exercise tests analyze VCO_2_, VO_2_, and RER through the use of indirect calorimetry; an attempt was made to utilize information generated from metabolic data while acknowledging the differences which will always persist between species.

The available equipment for exercise test in mice and human is another factor that was considered during development of the GXT_m_. In human tests, VCO_2_ and VO_2_ are measured each breath, whereas with mice, this information is generated from the gas exchange occurring inside the metabolic chamber which encloses the treadmill mice run on. Without the ability to calculate single breath values in running mice, the ventilatory rate cannot be calculated. Furthermore, the diffusion of gas from the chamber to the sensor elicits an approximate one-minute lag in mice testing. Thus, this must be accounted for data interpretation and analysis. Given that some of the biggest differences between mouse and human cardiac physiology occur in respect to heart rate, size, and oxygen requirements (reviewed in [60]), we recognized it was essential to rely on variables which were normalized to animal size and oxygen rates (RER, fuel substrate oxidation) to focus the similarities of comparative exercise physiology of mice and men.

Another difference between human and mouse testing included test termination. A shock grid wills maximal exertion attempts in mice; where as in humans, they run at their own volition. Accordingly, appropriate acclimation to treadmill testing must be completed in mice to reduce the physiological and psychological stress potentially associated with their initial introduction to shock. Outside of these limitations though, the adjustments we made to stage length and intensity, and the alterations we made to account for perpetual differences between mice and humans, allowed us to develop a method of exercise testing for mice that induced true VO_2max_ and generated a set of variables that were comparable to data acquired from human testing (AT, crossover, Lactate_delta_)

### The value of determining anaerobic threshold in mouse exercise testing

The field of exercise physiology has established that the relationship between VO_2_ and work rate diminishes in tests lasting less than 6 minutes or greater than 12 minutes [2]. Furthermore, they have shown that tests lasting over 12 minutes provide data that is impacted by skeletal muscle fatigue and orthopedic issues [2]. The PXT_m_ was composed of a large volume of submaximal work. During this exercise intensity, there is a decreased demand for oxygen and a reduction in the redistribution of blood from inactive to active tissues. This submaximal intensity delays time until maximum cardiac output, ventilation, and VO_2max_ [63]. In the PXT_m,_ this type of scenario occurs, as the cardiovascular system is not maximally stressed until later stages of the test. Accordingly, this test can be considered too long to specifically stress the cardiorespiratory system and its ability to withstand metabolic stress. Our data showed that the PXT_m_ is likely a superior test for assessing aerobic exercise capacity and aerobic endurance; whereas, the GXT_m_ is superior for assessing CVF.

A long test can be problematic if a researcher wants to report parameters beyond maximum run speed or duration, such as AT and crossover, to determine the cardiometabolic phenotype of a mouse. This limitation was observed in classic PXT_m_, as it was incapable of producing RER kinetics to determine AT. Typically AT can be identified by a nonlinear increase in minute ventilation [92]; however, this is not feasible for most researchers to calculate in mouse models during exercise [96]. In our GXT_m_ test we were able to use abrupt exponential increases in RER to determine the point at which AT occurred in both WT and dysfunctional models. These AT values derived in our GXT_m_ provided a sensitive measure for determining CVF in mice. Clinically, AT has been used in patients with cardiorespiratory disease to assess exercise tolerance [85]; however, the ability to derive AT from an exercise test has additional applications such as evaluating endurance performance, exercise prescription, and determining the effects of drugs on exercise tolerance (reviewed in [97]). Thus, the ability of the GXT_m_ to derive AT values highlighted its capability to generate novel noninvasive diagnostics and quantitative assessments of CVF in various mouse models.

### Metabolic crossover, an old human metabolic parameter with new applications in mouse testing

We found the GXT_m_ was capable of predicting AT based off of RER values, but it was also capable of determining the specific point of crossover from predominate lipid to carbohydrate oxidation during testing. This shift in fuel substrate utilization, known as the crossover concept [98], demonstrates that as relative VO_2_ and power output increase, there is a shift to predominate carbohydrate utilization. Thus, the shift from predominant of lipid oxidation to an increased dependence on muscle glycogen and blood glucose substrates [98] is intensity driven. This concept had been well established with methods such as radio-tracers, tissue metabolite sampling, stable isotopes, and indirect calorimetry in mammals and man [99] (reviewed in [98]). With the GXT_m_, the use of glycogen and glucose oxidation increased exponentially with exercise intensity and, therefore, allowed for crossover determination (Figs 6 and 7). It should be noted, as demonstrated in the data of a single WT mouse (Fig 2), that AT and crossover did not occur simultaneously in the GXT_m_ testing and were both difficult to interpret in PXT_m_ testing. The effect seen in the GXT_m_ could potentially be due to pyruvate dehydrogenase (PDH) mediated LA accumulation and aerobic substrate oxidation [100]. In working muscles, transformation of the pyruvate dehydrogenase complex (PDHc) to the active form (PDHa) is complete at approximately 80% VO_2max_ [101]; however, crossover occurs at approximately 65% percent of VO_2max_ [71]. With the GXT_m_, both crossover and AT were found around these approximations, with crossover occurring at 62–75% and AT occurring between 68–87% in mice. Regardless of these differences, the crossover obtained in our study with established model cardiovascular dysfunction was similar; yet significantly different than the healthy WT controls.

### Standardized methods for the functional assessment of cardiovascular fitness in mice

Without a gold standard *in vivo* exercise assay, reported data become both unreliable and difficult to reproduce between researchers. Previous mouse exercise assays have not considered both components of human exercise testing and the limitations of exercise testing in mice. However, as we have shown, certain testing conditions in mice allow for the reporting of cardio-metabolic parameters previously only reported in human testing. With the appropriate considerations to test design and differences between mouse and human physiology, tests like the GXT_m_ can serve as noninvasive, cost effective, methods to assess the cardio-metabolic phenotype of mice. Our data showed that the GXT_m_ was able to consistently provide data about the CVF of various models, and thus, could be used in the future to examine the effects of various treatments and therapeutics.

Alternative cardiac challenges using echocardiography [102] and cardiac magnetic resonance imaging (_c_MRI) [103] are popularized protocols to stress and test the cardiovascular system in mouse models; however, they are expensive and require animals to be anesthetized. Anesthesia prevents animal heart rates from achieving the true physiological responses to reagents and compromises cardiac output, a measure of blood being pumped by the heart per minute. Unlike these procedures, exercise assays have been shown to elicit a 2-fold increase in cardiac output [104] while avoiding limitations of anesthesia. Appropriate exercise testing and exercise prescription clearly have a place in the assessment and management of cardiovascular disease. Accordingly, exercise testing and prescription could carry a similar weight in mouse cardiovascular research if there was more standardization amongs the methods used to determine CVF in mice. If research done on mice is aimed at elucidating mechanisms of disease and therapies; then it is critical to apply tests that specifically test the CVF of mice when assessing cardio-metabolic function.

## Supporting Information

S1 File Contains supporting tables, figures, and notes. (DOCX)

## Nonstandard Abbreviations and Acronyms

APMHR: age-predicted maximal heart rate
AT: Anaerobic threshold
Casq2^-/-^: Cardiac calsequestrin deficient mice
CAD: Coronary artery disease
GXT_h_: Human graded maximal exercise test
GXT_m_: Mouse graded maximal exercise test
LA: Lactate
PDH: Pyruvate dehydrogenase
PXT_m_: Mouse progressive maximal exercise test
RER: Respiratory exchange ratio
RPE: rating of perceived exertion
RR: respiratory rate
VO_2_: Volume of oxygen consumed
VO_2max_: Maximal oxygen consumption
WT: Wild type
