# Supplementary material for: Graded Maximal Exercise Testing to Assess Mouse Cardio-Metabolic Phenotypes
Source: PLoS One. 2016 Feb 9;11(2):e0148010. doi: 10.1371/journal.pone.0148010 (PMC4747552; doi:10.1371/journal.pone.0148010)
Supplement: S1 File — (DOCX) [file pone.0148010.s001.docx]

**Supplemental Material**

**Graded Maximal Exercise Testing to Assess Mouse Cardio-Metabolic Phenotypes**

**Supplementary Tables**

**S1 Fig.** Differences between healthy wild type mouse and human graded maximal exercise data.

**S2 Fig.** Differences between single mouse and human graded maximal exercise test.

**Supplementary Tables**

**S1 Table.** Baseline metabolic parameters for mouse models

**S2 Table.** Animal acclimation to treadmill protocol.

**S3 Table.** Carbohydrate and fat oxidation values derived from RER values for calculation of crossover point.

**S4 Table.** Criteria for exercise test termination from the PXT_m_, GXT_h_, and GXT_m_.

**S5 Table.** Exercise end points parameters from the GXT_m_ and PXT_m_ in functional and dysfunctional animals.

**S6 Table.** RER and associated heat-derived values.

**S7 Table.** Exercise end points parameters from the GXT_m_ and PXT_m_ in WT C57bl/6J and FVB/NJ animals.

**S8 Table.** Exercise end points parameters from the GXT_m_ and PXT_m_ in FVB/NJ v. *Casq2^-/-^* animals.

**S9 Table.** Lactate concentration endpoints from the GXT_m_ and PXT_m_ in functional and dysfunctional animals.

**Supplementary Notes**

**S1 Text.** Gas exchange equations that can be derived by Oxymax software.

**S2 Text.** Formulas to construct exercise experiments (adapted from^2^).

**S3 Text:** Considerations for statistical analysis of calorimetry data.

**S4 Text.** Additional comparison of cardiovascular fitness in the control FVB/NJ compared to dysfunctional *Casq2^-/-^* mice.

**S5 Text:** New York Heart Association classifications of heart failure based on patient symptoms.

**S1 Fig. Differences between healthy mouse and human graded maximal exercise data.**

**

**

**S1 Fig. Differences between healthy mouse and human graded maximal exercise data.** Averaged values obtained in 15 second (sec) intervals from WT mice (*n*=7) performing both the PXT_m_ (left panels) and GXT_m_ tests (middle panels); as well as humans performing the GXT_h_  (*n*=6, right panels). (**a)** VO_2_ (red line) and VCO_2_ (blue line) values intersect at VO_2max_ (black arrow) in the PXT_m,_ **(b)** GXT_m_ and **(c)** GXT_h_. The pattern seen in mice was similar to healthy human test averages; however, levels of VO_2_ and VCO_2_ are lower. **(d)** Using the same tests displayed above are RER (orange) values in the PXT_m_, **(e)** GXT_m_, and **(f)** GXT_h_. An abrupt exponential increase in RER indicates anaerobic threshold (AT, black dotted line). **(g)** Crossover is determined from plotting carbohydrate (dark blue line) and fat (light blue line) oxidation during testing on the PXT_m_, **(h)** GXT_m_ and **(i)** GXT_h_.

**S2 Fig. Kinetics and parameters from single GXT_m_, and GXT_h_ tests.**

**

**

**S2 Fig. Kinetics and parameters from single GXT_m_, and GXT_h_ tests.** **(a)** During a GXT_m_ mice exhibit increases in VO_2_ (dark green line) and VCO_2_ (light green line), which intersect at VO_2max_. **(b)** This same pattern is seen (middle, right) with humans; however, levels of VO_2_ (dark grey line) and VCO_2_ (light grey line) are lower. **(c)** Both mice, and men **(d)** have sudden abrupt exponential increases in RER during testing, which can be used to indicate the shift from aerobic (light area) to anaerobic (dark area) metabolism. **(e)** By plotting both carbohydrate oxidation and fat oxidation during testing in mice and **(f)** men, the crossover point.

**S1 Table: Baseline metabolic parameters for mouse models.**

| **Stage** | **Weight**  (g) | **Basal VO_2_**  (ml/kg/min) | **Duration**  (mmol/L) |
| --- | --- | --- | --- |
| WT- C57BL/6J | 28.15 ± 1.46 | 73.14 ± 11.56 | 2.44 ± 1.22 |
| Obese - C57BL/6J | **45.42 ± 2.66** | **55.24 ± 11.84** | 2.89 ± 1.08 |
| *Casq2^-/-^* | **25.72 ± 0.87** | **86.89 ± 7.32*** | 3.56 ± 1.79 |
| WT- FVB/NJ | 29.23 ± 1.84 | 74.10 ± 9.20 | 2.03 ± 0.86 |

Data are shown as mean ±SD. Bold indicates is significant difference between genotypes at the alpha = .007 level (MANOVA, multiple comparisons Tukey HSD, WT, *n* = 7; *Casq2^-/-^,* *n* = 4, WT-FVB/NJ, *n* = 4; WT-Obese, *n = 11*). Asterisk indicates difference between the *Casq2^-/-^* and FVN/NJ mice (Student’s t-Test, alpha = .05 level).

**S2 Table: Animal acclimation to treadmill protocol.** Shock grid was activated (3 Hz and 1.5 mA). Acclimation was preformed three times with 60 hours of recovery between training sessions.

| **Stage** | **Speed**  (meter/min) | **Elevation**  (%grade) | **Duration**  (min) |
| --- | --- | --- | --- |
| **1** | 0 | 0 | 3 |
| **2** | 6 | 0 | 5 |
| **3** | 9 | 0 | 2 |
| **4** | 12 | 0 | 2 |

**S3 Table: Carbohydrate and fat oxidation values derived from RER values for calculation of crossover point.** These values represent nonprotein RER values per liter of oxygen utilized refs. :

Lusk G. The elements of the science of nutrition. 3d ed. Philadelphia,: W.B. Saunders; 1923. 641 p. p.

Peronnet F, Massicotte D. Table of nonprotein respiratory quotient: an update. Canadian journal of sport sciences = Journal canadien des sciences du sport. 1991;16(1):23-9. PubMed PMID: 1645211.

| **RER** | **%CHO** | **%FAT** | **RER** | **%CHO** | **%FAT** |
| --- | --- | --- | --- | --- | --- |
| **0.7** | 0.0 | 100.0 | **0.86** | 55.8 | 47.6 |
| **0.71** | 1.4 | 98.6 | **0.87** | 52.4 | 44.2 |
| **0.72** | 4.8 | 95.2 | **0.88** | 59.2 | 40.8 |
| **0.73** | 8.2 | 91.8 | **0.89** | 62.6 | 37.4 |
| **0.74** | 11.6 | 88.4 | **0.90** | 66.0 | 34.0 |
| **0.75** | 15.0 | 85.0 | **0.91** | 69.4 | 30.6 |
| **0.76** | 18.4 | 81.6 | **0.92** | 72.8 | 27.2 |
| **0.77** | 21.8 | 78.2 | **0.93** | 76.2 | 23.8 |
| **0.78** | 25.2 | 74.8 | **0.94** | 79.6 | 20.4 |
| **0.79** | 28.6 | 71.4 | **0.95** | 83.0 | 17.0 |
| **0.80** | 32.0 | 68.0 | **0.96** | 86.4 | 13.6 |
| **0.81** | 35.4 | 64.6 | **0.97** | 89.8 | 10.2 |
| **0.82** | 38.8 | 61.2 | **0.98** | 93.2 | 6.8 |
| **0.83** | 42.2 | 57.8 | **0.99** | 96.6 | 3.4 |
| **0.84** | 45.6 | 54.4 | **1.00** | 100.0 | 0.0 |
| **0.85** | 49.0 | 51.0 |  |  |  |

Analysis of the oxidation of mixtures of carbohydrate and fat (derived from Luck et al, 1923). Formulas which Oxymax software utilizes, as well as fuel substrate charts, are based off calculations were derived using direct animal (dog) calorimetry, radioactive isotope labeling of isotopes 12 and 13 of carbon, and urinary nitrogen excretion measurements.

**S4 Table:** **Criteria for exercise test termination from the PXT_m_, GXT_h_, and GXT_m_.** In human testing significant increases in lactic acid are described as between 8 to 10 mmol/L, but variable [4].

| **Test End Points** | **RER** | **VO_2_ Plateau** | **Lactic Acid Conc.** | **Exertion** |
| --- | --- | --- | --- | --- |
| **PXT_m_** | RER ≥ 1.0 | **No plateau of O_2_ required** | **No LA measure required** | 5 sec of continuous contact with shock grid |
| **GXT_h_** | RER ≥ 1.1 | Plateau/no change of O_2_ uptake with increasing workload | Significant ↑ in post exercise LA concentrations | **RPE ≥ 9 on scale 1-10** |
| **GXT_m_** | RER ≥ 1.0 | Plateau/no change of O_2_ uptake with increasing workload | Significant ↑ in post exercise LA concentrations | 5 sec of continuous contact with shock grid |

**S5 Table: Exercise end points parameters from the GXT_m_ and PXT_m_ in functional and dysfunctional animals.**

| **Genotype** | **Relative VO_2max_** | **Max Run Speed** | **Exhaustion** | **LA_delta_** |
| --- | --- | --- | --- | --- |
|  | (ml/kg/min) | (m/m) | (min) | (mmol/L) |
| **PXT_m_** |  |  |  |  |
| WT | 127.06 ± 6.76 | 26.50 ± 4.43 | 26.75 ± 4.48 | 4.08 ± 1.38 |
| Obese | **77.64 ± 8.30** | 22.75 ± 1.75 | 23.34 ± 1.63 | 5.15 ± 3.19 |
| *Casq2^-/-^* | 107.00 ± 8.66 | 32.33 ± 2.08 | 32.82 ± 1.66 | 2.50 ± 2.52 |
| **GXT_m_** |  |  |  |  |
| WT | 134.84 ± 5.80 | 27.29 ± 1.70 | 14.68 ± 1.78 | 6.63 ± 2.17 |
| Obese | **88.52 ± 7.17** | **21.64 ± 2.06** | **10.05 ± 0.84** | 4.51 ± 3.08 |
| *Casq2^-/-^* | **107.77 ± 11.80** | **23.75 ± 0.50** | **11.31 ± 0.55** | **9.32** ±**1.53** |

Values are based on observed means ±SD. Bold indicates is significant difference between genotypes for either the PXT_m_ or GXT_m_ at the alpha = .007 level (MANOVA, multiple comparisons Tukey HSD of dysfunctional mice compared to WT).

**S6 Table:** **RER and associated heat-derived values.**

| **RER** | **Heat/Liter O_2_ (kcal)** | **RER** | **Heat/Liter O_2_ (kcal)** |
| --- | --- | --- | --- |
| **0.707** | 4.6862 | **0.900** | 4.9226 |
| **0.750** | 4.7387 | **0.950** | 4.9847 |
| **0.800** | 4.8008 | **1.000** | 5.0468 |
| **0.850** | 4.8605 |  |  |

**S7 Table: Exercise end points parameters from the GXT_m_ and PXT_m_ in WT C57BL/6J and FVB/NJ animals.**

| **Genotype** | **Relative VO_2max_** | **Max Run Speed** | **Exhaustion** | **LA_delta_** |
| --- | --- | --- | --- | --- |
|  | (ml/kg/min) | (m/m) | (min) | (mmol/L) |
| **PXT_m_** | | | | |
| WT | 127.06 ± 6.76 | 26.50 ± 4.43 | 26.75 ± 4.48 | 4.08 ± 1.38 |
| FVB/NJ | 119.73 ± 6.35 | **39.50 ± 3.79** | **39.89 ± 3.77** | 3.23 ± 1.39 |
| **GXT_m_** |  |  |  |  |
| WT | 134.84 ± 5.80 | 27.29 ± 1.70 | 14.68 ± 1.78 | 6.63 ± 2.17 |
| FVB/NJ | **119.13 ± 9.56** | 29.25 ± 2.5 | 16.56 ± 2.73 | 3.23 ± 1.39 |

Based on observed means ±SD. Bold indicates is significant at the alpha = .05 level in WT v. FVB/NJ on a given test (Student’s t-Test).

**S8 Table: Exercise end points parameters from the GXT_m_ and PXT_m_ in FVB/NJ v. *Casq2^-/-^* animals.**

| **Genotype** | **Relative VO_2max_** | **Max Run Speed** | **Exhaustion** | **LA_delta_** |
| --- | --- | --- | --- | --- |
|  | (ml/kg/min) | (m/m) | (min) | (mmol/L) |
| **PXT_m_** |  |  |  |  |
| FVB/NJ | 119.73 ± 6.35 | 39.50 ± 3.79 | 39.89 ± 3.77 | 3.23 ± 1.39 |
| *Casq2^-/-^* | 107.00 ± 8.66 | **32.33 ± 2.08** | **32.82 ± 1.66** | 2.50 ± 2.52 |
| **GXT_m_** |  |  |  |  |
| FVB/NJ | 119.13 ± 9.56 | 29.25 ± 2.5 | 16.56 ± 2.73 | 3.23 ± 1.39 |
| *Casq2^-/-^* | 107.77 ± 11.80 | **23.75 ± 0.50** | **11.31 ± 0.55** | **9.32 ±1.53** |

Based on observed means ±SD. Bold indicates is significant at the alpha = .05 level in FVB/NJ v. *Casq2^-/-^* on a given test (Student’s t-Test).

**S9 Table: Lactate concentration endpoints from the GXT_m_ and PXT_m_ in functional and dysfunctional animals.**

| **Genotype** | **Post LA** (mmol/L) | **LA_delta_** (mmol/L) |
| --- | --- | --- |
|  |  |  |
| **PXT_m_** |  |  |
| WT | 6.53 ± 1.49 | 4.08 ± 1.38 |
| Obese | 7.31 ± 2.18 | 5.15 ± 3.19 |
| *Casq2^-/-^* | 7.60 ± 1.04 | 2.50 ± 2.52 |
| **GXT_m_** |  |  |
| WT | 9.06 ± 2.20 | 6.63 ± 2.17 |
| Obese | 8.12 ± 2.78 | 4.51 ± 3.08 |
| *Casq2^-/-^* | 10.50 ± 2.05 | **9.32 ± 1.53** |

Based on observed means ± SD. Bold indicates is significant difference between genotypes for either the PXT_m_ or GXT_m_ at the alpha = .007 level (MANOVA, multiple comparisons Tukey HSD of dysfunctional mice compared to WT controls).

**S1 Text: Gas exchange equations that can be derived from Oxymax software.**

All treadmill testing was done in a metabolic modulator treadmill (Columbus Instruments, Columbus, OH, USA). This treadmill (24" exercise belt, speeds from 0 m/m to 99.9m/m, inclination -10˚ to 25˚, adjustable shock grid from 0.35mA to 1.5mA) is enclosed in an air-tight isolated chamber (29"L x 27"W x 17.5"H) allowing it to function as an open circuit indirect calorimeter. Thus, the metabolic modulator treadmill functions as an indirect calorimeter. With the metabolic modulator treadmill, oxygen consumption (VO_2_) and carbon dioxide expiration (VCO_2_) are calculated using Oxymax software. This software is dependent on accurate measurements of gas concentrations and flow, and thus needs to be calibrated prior to all experiments involving gas exchange assessments.

To make these calculations during testing the Oxymax software collects values of either the mass of air at chamber input per unit of time (Vi) or the mass of air at chamber output per unit of time (Vo) and then predicts the alternate flow (under the assumption that nitrogen is equal in the input and output portion of the chambers, and does not take part in respiratory gas exchange)*:

Vi = Mass of air at chamber input per unit time

O_2_i = Oxygen fraction in Vi

CO_2_i = Oxygen fraction in Vi

Vo = Mass of air at chamber output per unit time

O_2_o = Carbon Dioxide fraction in Vo

CO_2_o = Carbon Dioxide fraction in Vo

From those values VO_2_ and VCO_2_ are calculated by Oxymax:

VO_2_ = ViO_2_i i-VoO_2_o

VCO_2_= VoCO_2_o -ViCO_2_i

From VO_2_ and VCO_2_ values collected, the software, Oxymax, then determines respiratory exchange ratio (RER).

RER = VCO_2_/ VO_2_

From RERs between 0.7 and 1.0, carbohydrate and fat oxidation per liter of oxygen used can be calculated (S5 Table) as can the percent of carbohydrates and fat oxidized per minute.

CHO (g/min) = -3.226 * VO_2_(L/min) + 4.585*VCO_2_(L/min); Peronnet et al, 1991

FAT (g/min) = 1.695 * VO_2_(L/min) - 1.701* VCO_2_(L/min); Peronnet et al, 1991

For RER values between 0.7 and 1.0, 4.686 to 5.047 Kcal/Liter O_2_ (Heat) is available (from ref.: McLean JA, Tobin G. Animal and human calorimetry. Cambridge Cambridgeshire ; New York: Cambridge University Press; 1987. xiii, 338 p. p.).

*Detailed information about calculation information, and equations shown here, are included in Columbus Instrument’s Oxymax Software user manuals.

**S2 Text:** **Formulas to construct exercise experiments (adapted from [4]) RER, heat-derived values, metabolic equivalents (METS, S9 Table), and intensities for exercise experiments.**

To calculate the caloric value, the following formula can then be applied using RER values:

CV (kcal/liter of O_2_) = 3.815 + 1.232 x RER

Calculating the caloric value allows for the derivation of energy expenditure (heat) of a mouse during exercise:

Heat (kcal/hour) = CV(kcal/liter of O_2_) x VO_2_ (ml/kg/hr)

*Note, in this manuscript,VO_2_ is reported as ml/kg/min

Additionally, VO_2_ values can be used to derive metabolic equivalents (METs)

MET = VO_2_/kg ÷ 3.5

Those VO_2_ values can then be used to derive metabolic equivalents (METs) to prescribe intensities to train animals at (adapted from [4]). MET_max_ indicates maximum METS calculated at VO_2max_ and % MET_max_ indicates the percentage of MET_max_ to exercise at.

| **Intensity** | **% of MET_max_** |
| --- | --- |
| Very Light | <30-35% |
| Light | 30-50% |
| Moderate | 45-65% |
| Hard | 65-85% |
| Very Hard | ≤85% |

VO_2max_ is critical to determine, as workload can then be quantified as METS (metabolic equivalents), and adapted from ACSM recommendations for general and special populations to prolonged exercise experiments in mice. When METS are used in combination with various other metrics like AT, more specific recommendations can be made ( refs: [3, 9] and Pina IL, Madonna DW, Sinnamon EA. Exercise test interpretation. Cardiology clinics. 1993;11(2):215-27. Epub 1993/05/01. PubMed PMID: 8508448). METs can be used to prescribe intensities in long-term exercise experiments. Additionally, in some models with cardiovascular and/or skeletal muscle limitations, exercise that is too great in intensity may elicit maladaptation, as the stress is to great for the organism to overcome. In other scenarios, intensity might not enough to elicit an adaptation. Exercise, which is a stressor, thus must be great enough to disrupt homeostasis, if the intent is to bring about an adaption. Accordingly, METS can be divided into appropriate ranges of exercise intensities for animals to train at provided information about VO_2max_ is collected [4].

**S3 Text: Considerations for statistical analysis of calorimetry data**

One of the longest debated topics ( refs.: Kleiber M. The fire of life; an introduction to animal energetics. New York,: Wiley; 1961. 454 p. p.; Kleiber M. Body size and metabolic rate. Physiological reviews. 1947;27(4):511-41. PubMed PMID: 20267758.) in the study and analysis of mouse energy expenditure, at rest and during activity, is whether the data should be normalized to body weight or body composition. To date, the most utilized method of analysis is to divide oxygen consumption or energy expenditure by body weight. However, alternative methods where weight is used as a covariate are also utilized (reviewed in Tschop MH, Speakman JR, Arch JR, Auwerx J, Bruning JC, Chan L, et al. A guide to analysis of mouse energy metabolism. Nature methods. 2012;9(1):57-63. doi: 10.1038/nmeth.1806. PubMed PMID: 22205519; PubMed Central PMCID: PMC3654855). Here, we discuss here the types of analyses and their limitations. We also provide alternative data analysis for the data presented in this paper as an example.

Over the years, there has been a trend to normalize metabolic data by lean mass, rather than body weight. This is problematic though, as organs like skeletal muscle, brown adipose tissue, and white adipose tissue have differential energy requirements and effects on whole body metabolism. In line with this notion, it could be, and has been proposed, that researchers should divide energy expenditure and oxygen consumption by a divisor which incorporates each type of tissue’s respective metabolic effects (reviewed in Tschop et al., 2012).

Considering that weight could have confounding results on metabolic data, some researches have switched to using analysis of covariance (ANCOVA) (refs. Tschop et al., 2012., and Allison DB, Paultre F, Goran MI, Poehlman ET, Heymsfield SB. Statistical considerations regarding the use of ratios to adjust data. International journal of obesity and related metabolic disorders : journal of the International Association for the Study of Obesity. 1995;19(9):644-52. PubMed PMID: 8574275). ANCOVA is a general linear model that combines analysis of variance (ANOVA) and regression analysis. ANCOVA can be used to determine if there are differences between independent groups following the adjustment for a variable or variables (the covariate or covariates) that may confound results (Rutherford A, ebrary Inc. Introducing Anova and Ancova a GLM approach. London: SAGE,; 2000). In situations where one or more of the variables being examined is affecting the results, ANCOVA is appropriate, so long as all nine of its assumptions are met. Often times, the studies that use ANCOVAs have a large sample size (*n ≥* 20 per group), which is required to reach statistical power. Statistical power is critically important to correctly detecting differences and interpreting data. Others have suggested the impracticality of using this type of analysis though (Butler AA, Kozak LP. A recurring problem with the analysis of energy expenditure in genetic models expressing lean and obese phenotypes. Diabetes. 2010;59(2):323-9. doi: 10.2337/db09-1471. PubMed PMID: 20103710; PubMed Central PMCID: PMC2809965), as few animal studies have utilized large enough sample sizes to justify ANCOVA when using exercise assays to phenotype groups of mice [23, 58, 63, 64, 66, 69]. Muscle, brown fat, and white fat mass should be acknowledged for their differential effects on calorimetry measures. Thus, if an ANCOVA is to be used as the method of statistical analysis, then brown fat, white fat, and muscle mass should each be treated as separate covariates. To do such a type of analysis though, fat masses and muscle mass need to be accounted for. Both fat mass and muscle mass have individual and differential effects on calorimetry data, specifically VO_2max_ during exercise testing (Tompuri T, Lintu N, Savonen K, Laitinen T, Laaksonen D, Jaaskelainen J, et al. Measures of cardiorespiratory fitness in relation to measures of body size and composition among children. Clinical physiology and functional imaging. 2015;35(6):469-77. doi: 10.1111/cpf.12185. PubMed PMID: 25164157). In this publication, fat mass has been shown to be a better predictor of relative VO_2max_ compared to test performance and lean mass represents the oxygen-processing capabilities of a mouse. Thus, these types of mass represent two, separate, informative, traits that can best be summarized as “fitness” from oxygen-processing (VO_2max_/muscle mass) and metabolic (VO_2max_/ fat mass) perspectives. Of note, the addition of multiple covariates introduces the need for both increased sample sizes to reach statistical power and methods to determine the mass of each tissue. Tissue collection is normally done by destructive methods like animal sacrifice or cost-prohibitive, *in vivo,* methods like magnetic resonance spectroscopy, dual-energy x-ray absorptiometry, and magnetic resonance imaging. Thus, all things should be considered prior to running ANCOVA analysis to correctly interpret the data.

Below is the comparison of our results when adjusted for weight (ANCOVA) and unadjusted (ANOVA). Our results were rather similar between the adjusted and unadjusted analysis, but some differences did occur:

We compared the results of the calorimetry data by performing ANOVAs and ANCOVAS, so we could understand how treating weight as a covariant affected the data. Without adjusting for weight (ANOVA), there were significant differences across genotypes, regardless of test type, for the following calorimetry variables: relative VO_2max_ max (*F*(2, 37) = 125.1, *p* < .001), absolute VO_2max_ (*F*(2, 37) = 350.122, *p* < .001), delta VO_2_ (*F*(2, 37) = 15.661, *p* < .001), and % of test where ventilatory threshold occurred (*F*(2, 34) = 8.381, *p* = .001). In general, wild type mice had higher values of relative and absolute VO_2max_, and delta VO_2_, and lower values of % of test where ventilatory threshold compared to obesity and cardiac mice. Non calorimetry variables such as max speed (*F*(2, 37) = 25.016, *p* < .001) and time until exhaustion *F*(2, 37) = 26.835, *p* < .001) were also significantly changed between genotypes, with wild type mice having higher values compared to obese and *Casq2-/-*  mice.

Additionally, there were significant differences between GXT_m_ and PXT_m_ across all mice genotypes for the relative VO_2max_ (*F*(1, 37) = 1.403, *p* = .030), with the PXT_m_ yielding lower relative VO_2max_  compared to the GXT_m_. Non calorimetry variables such as max speed (*F*(1, 37) = 13.78, *p* = .001), time until exhaustion (*F*(2, 37) = 513.846, *p* < .001), post lactate (*F*(1, 37) = 6.289, *p* = .018), and delta lactate (*F*(2, 37) = 8.442, *p* = .007) were also changed between tests, with the PXT_m_ yielded lower relative VO_2max ,_ post lactate levels, and delta lactate levels, as well as higher max speed and time until exhaustion compared to the GXT_m_.

We then performed ANCOVAs, treating weight as the covariant. When adjusting for weight, we still observed significant differences in the calorimetry data across genotypes for relative VO_2max_ (*F*(2, 37) = 31.525, *p* < .001), absolute VO_2max_ (*F*(2, 37) = 24.637, *p* < .001), delta VO_2_ (*F*(2, 37) = 11.328, *p* < .001) and % of test where ventilatory threshold occurred (*F*(2, 34) = 4.096, *p* = .001) with wild type mice having higher relative and absolute VO_2max_ values, higher delta VO_2_, and lower % of test where ventilatory threshold occurred compared to obesity and cardiac mice. Additionally, there was a significant difference between tests when looking across all genotypes for max speed (*F*(1, 37) = 13.361, *p* = .001), time until exhaustion (*F*(1, 37) = 498.738, *p* > .001), post lactate (*F*(1, 37) = 6.4, *p* = .017), and delta lactate (*F*(1, 37) = 8.617, *p* = .006). PXT_m_  higher max speed and time until exhaustion, as well as lower post lactate and delta lactate compared to the GXT_m_. There were interaction effects between test and genotype for max speed and time until exhaustion, suggesting that differences in these values between tests was depending upon the mouse genotype.

Given the sample size of our study, the power was not sufficient to meet all assumptions to justify running the ANCOVA or to generate outcome statements. Additionally, treating weight as a covariant, rather than fat mass and muscle mass as two separate covariates, may have impacted how the ANCOVA results could be interpreted.

**S4 Text: Additional comparison of cardiovascular fitness in the control FVB/NJ compared to dysfunctional cardiac *Casq2^-/-^* mice.**

We presumed the superior performance of the *Casq2^-/-^* in the PXT_m_ could be the result of either 1) the test not being intense enough to elicit intensity above the ischemic threshold or 2) the *Casq2^-/-^* mice having superior submaximal endurance exercise capacities when working below the ischemic threshold. In humans with the CASQ mutation, exercise tests are used as a diagnostic test for catecholaminergic polymorphic ventricular tachycardia [107]. However, if a test is not intense enough it could have limited diagnostic capability.

The FVBN/J control strain has documented improved performance on endurance exercise testing in measurements for duration, distance and work performance [87, 88]. Accordingly, we performance all tests in the FVB/NJ to compare to the *Casq2^-/-^* as an alternate control (S6 and S7 Tables). We found the FVBN/J model had superior fitness to WT C57Bl/6 (Student’s t-Test, *p* = 0.05) with increased max speed and time till exhaustion on the PXT_m_. Interestingly, only the GXT_m_, was able to show that the FVB/NJ had increases in relative VO_2max_, VO_2delta_, post LA, and LA_delta_. This indicated the GXT_m_ had superior sensitivity compared to the PXT_m_, as a result to specific stress to the cardiovascular system without long duration capable of inducing additional fatigue to the musculoskeletal system.

When comparing the performance of the *Casq2^-/-^* to the FVB/NJ on the GXT_m_, there was a significant difference in max speed, time until exhaustion, VO_2delta_ (Student’s t-Test, *p* < .05) and with the PXT_m_ there was a difference in speed, VO_2delta_, and post LA (student’s t-Test, *p* = .05) indicating that there was no difference in time until exhaustion. For the FVB/NJ in a test to test comparison, the PXT_m_ resulting in a significantly lower LA_delta_ (student’s t-Test, *p* < .01; 3.22 ± 1.38 mmol/L, PXT_m_; 6.96 ± 1.35 mmol/L, GXT_m,_ S3-5 Tables) indicating that even when using a control strain with superior fitness, the GXT_m_ was superior in eliciting a significant increase in LA concentrations; which is an end point for a positive VO_2max_ test [4].

**S5 Text: New York Heart Association classifications of heart failure based on patient symptoms.**

The New York Heart Association (NYHA) has a functional classification system that doctors use to classify a patient’s heart failure according to symptom severity.

| **Class** | **Patient Symptoms** |
| --- | --- |
| I | No limitation of physical activity. Ordinary physical activity does not cause undue fatigue, palpitation, dyspnea (shortness of breath). |
| II | Slight limitation of physical activity. Comfortable at rest. Ordinary physical activity results in fatigue, palpitation, dyspnea (shortness of breath). |
| III | Marked limitation of physical activity. Comfortable at rest. Less than ordinary activity causes fatigue, palpitation, or dyspnea. |
| IV | Unable to carry on any physical activity without discomfort. Symptoms of heart failure at rest.  If any physical activity is undertaken, discomfort increases. |
